# Understanding the home range characteristics of the first naturally bred pair of crested ibis(*Nipponia nippon*) released into the natural habitat

**DOI:** 10.1186/s40850-024-00220-0

**Published:** 2024-11-29

**Authors:** Soodong Lee, Chunghyeon Oh, Bonggyo Cho, Youngsub Han

**Affiliations:** 1 Department of Landscape Architecture, Gyeongsang National University, 33 Dongjin-ro, Jinju-si, Gyeongsangnam-do 52725 Korea; 2 Department of Urban System Engineering, Gyeongsang National University, 33 Dongjin-ro, Jinju-si, Gyeongsangnam-do 52725 Korea; 3 Forest Ecology & Restoration Center, Korea Forest Conservation Association, 28 Munjeong-ro, Seo-gu, Daejeon, 35261 Korea

**Keywords:** Nest environment, Home range analysis, First breeding, Reintroduction, Nest, GPS tracking, KDE, MCP

## Abstract

**Background:**

The crested ibis, a species that relies on wetland ecosystems for survival, was once found throughout East Asia but has declined to near extinction in Korea, Russia, and Japan, except China. Artificial propagation of seven individuals found in Yangxian, Shaanxi Province, China has resulted in a stable population. Furthermore, South Korea and Japan are working on restoring populations through donations from China. Artificial propagation began in 2008, and in 2019, 40 individuals born between 2014 and 2018 were released into the natural habitat for the first time. We conducted this study to analyze the habitat environment, home range, and habitat usage patterns of a 2016-born male and a 2017-born female who attempted to reproduce naturally for the first time.

**Results:**

After forming a breeding pair on April 3, 2020, the pair made two breeding attempts, built a nest in *Pinus densiflora*, and succeeded in hatching the chicks, but failed to raise them. The home range analysis showed that the area was 1.777–2.425 km² for MCP 100%, and 0.347–2.085 km² for 95% KDE. Meanwhile, the core habitat ranged from 0.007 to 0.296 km² (KDE 50%), indicating differences depending on the time of year and the individual being studied. Breeding pairs were estimated to spend over 50% of their recorded occurrences within 50 m during nesting for incubation, resting, and other activities. They mainly used in paddy fields, but from April to June, when onions and garlic were growing, they searched for food in fields, cemeteries, reservoirs, and other areas.

**Conclusion:**

Breeding pairs have increasingly become more active near the nest, and Changnyeong-gun, where they were released, has large agricultural land suitable for crested ibis habitat. However, there is a problem that during the breeding season from April to June, most paddy fields are maintained as garlic and onion fields, which are then converted back for rice cultivation from May to June through double-cropping. Accordingly, for stable laying and rearing, it is necessary to contemplate how to maintain rice paddies, which serve as feeding grounds in the core habitats.

## Background

Wetlands, vital for species diversity and providing critical habitats for wild birds, have globally declined by 64–71% since the 20th century, posing severe threats to biodiversity and local communities dependent on these ecosystems [1–3]. Effective conservation goals at both biological and regional levels are essential to mitigate this loss [4, 5]. Large endangered species, such as storks, cranes, and crested ibises, are particularly vulnerable to these changes and require targeted conservation efforts to maintain viable populations.

The crested ibis(*Nipponia nippon*) populations began to decline sharply due to habitat loss and environmental changes from agricultural mechanization and development. In the United States, cumulative wetland loss since European settlement has reached ≥ 30%, and ≥ 80% overall since the 18th century [6]. Similarly, China has lost ≥ 30% of coastal wetlands and 25% of freshwater wetlands over the past 35 years [7], and Japan’s ibis populations dwindled to just five individuals by 1980 due to hunting and habitat destruction [8, 9].

Conservation efforts, including artificial propagation initiated in 1981, have helped stabilize ibis populations, though concerns about inbreeding depression due to low genetic variation persist [5, 8–11]. Ensuring the long-term stability of these populations necessitates extensive, long-term monitoring and data collection [12, 13]. The last known wild population of crested ibises in China was found near the Qinling Mountains, highlighting the need for habitats with suitable nesting trees and feeding areas [14, 15]. Sustainable development and conservation measures are required to balance habitat stability with local development, including policy support, habitat improvement, and community involvement [10, 15].

The reintroduction programs in Japan and South Korea, utilizing birds donated from China, have shown promise in restoring local ibis populations [10, 16]. In Korea, after the species went extinct in the wild in 1979, a pair imported from China in 2008 led to successful wild releases starting in 2019 [17]. Among the released individuals, the first breeding pair formed in 2020 and established their first nest at the edge of a forest in Mogok-ri, Ibang-myeon, Changnyeong-gun, Gyeongsangnam-do. They attempted to incubate their eggs but failed due to interference from weasels, their natural predators. Subsequently, they built a second nest in the nearby forest periphery but were again unsuccessful. This highlights the global significance of the restoration efforts for the crested ibis and underscores the need for comprehensive breeding and habitat management strategies.

The purpose of this study is to analyze the breeding behavior patterns of the crested ibis using GPS tracking and field surveys. Specifically, we aim to; (1) Compare the observed behavior patterns with those documented in previous studies and investigate any differences between the first and second nesting attempts to provide important baseline data for follow-up research. (2) Propose conservation and future management strategies based on the findings, offering insights that can be applied to the ongoing management of crested ibis populations.

## Materials and methods

### Study area

**Fig. 1:**
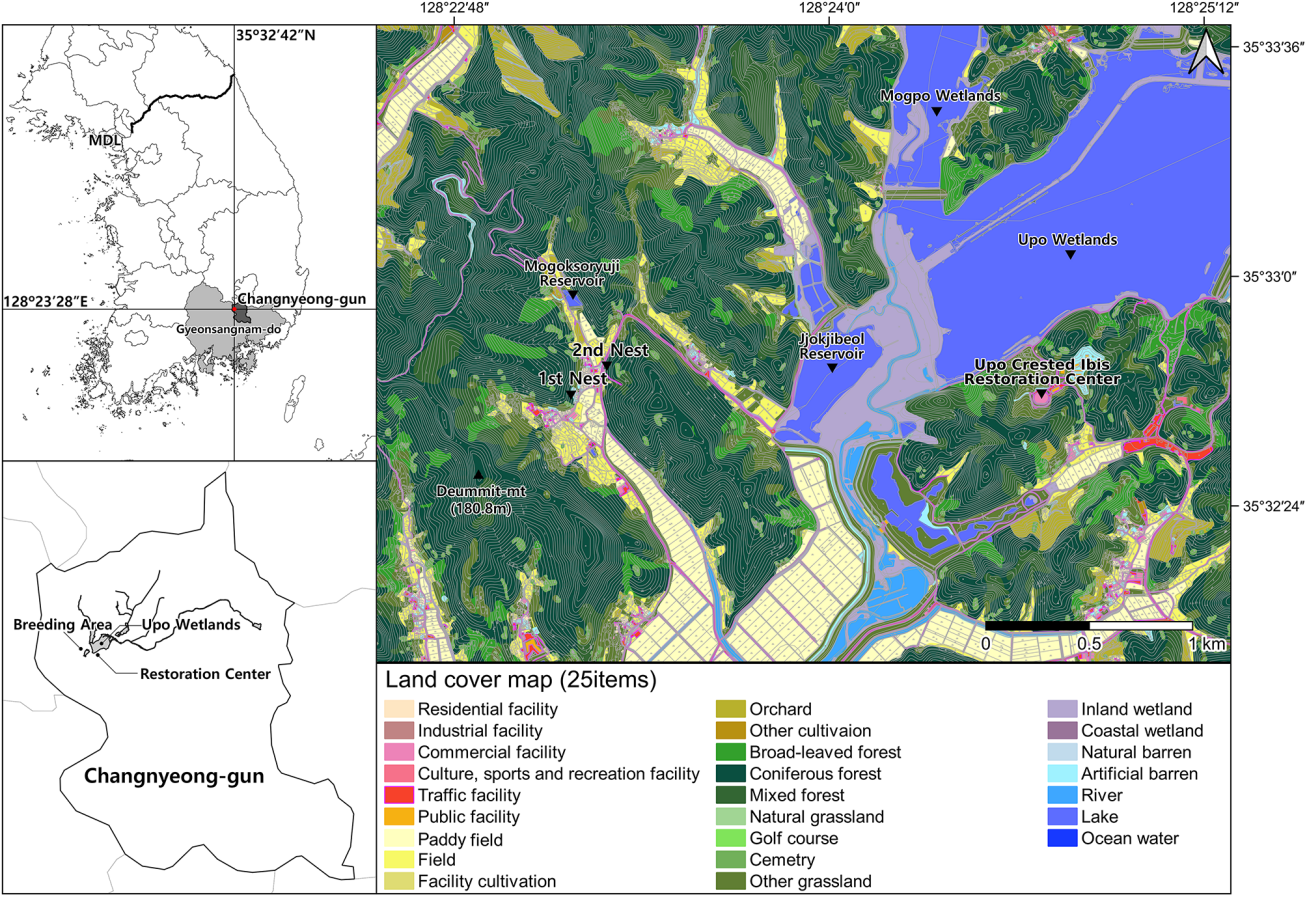
Locations of the nesting sites of crested ibis. The 25 items used in the land cover map were reclassified as appropriate for this study in compliance with the guidelines for preparing land cover maps of the Republic of Korea. A detailed explanation of the classification criteria can be found in the research methods

The study was conducted at the Upo Crested Ibis Restoration Center(hereafter, “UCIRC”) located at Sejin-ri, Yueo-myeon, Changnyeong-gun, Gyeongsangnam-do, and the Mogok reservoir area(35°32’24”-35°33’36” N, 128°22’48”-128°25’12” E), and Fig. 1 shows this. The UCIRC has been breeding crested ibises imported from China and releasing the young ones into the wild since 2019. Adjacent to the UCIRC is the Upo Marsh, the largest inland wetland in South Korea and a designated Ramsar site, covering 2.31 million km². Surrounding the area are extensive agricultural lands and forests featuring naturally occurring coniferous trees [18].

Most agricultural lands in this region are cultivated with garlic and onion from April to June, coinciding with the breeding season for the crested ibis. These lands are then converted to paddy fields from May to June after the crops are harvested, following a two-crop farming system similar to that practiced throughout Changnyeong-gun [19, 20]. In 2020, Changnyeong-gun had 7,288 ha of paddy fields and 2,732 ha of fields, totaling 10,020 ha, of which 3,738 ha (37%) were planted with garlic and onion [21]. Thus, only a small portion of the area is maintained as year-round paddy fields.

### Research subject

Artificially hatched young ibises were released into the wild after being fitted with GPS transmitters and color bands, following a training program for wild adaptation. In Korea, 40 individuals (27 males and 13 females) born between 2014 and 2018 were released into the wild for the first time on May 22, 2019 [22]. Subsequently, an additional 40 individuals were released on May 28, 2020, May 6, 2021, and October 14, 2021. The average weight just before release was 1.45 kg for males and 1.6 kg for females.

The locations and natural adaptations of the released individuals were determined by surveying the area around the coordinate points received by the attached radio tracking devices or through public reports, sightings, and requests for rescue. Individuals were identified via visual observation, binoculars or field scopes, photography, and videography to verify band numbers and identify their behavioral patterns. In the process of monitoring the adaptation and behavioral patterns of the identified individuals, a male (UPO1918) born in 2016 and a female (UPO1938) born in 2017, among the individuals first released in 2019, was confirmed to form the first breeding pair in Korea on April 3, 2020, 318 days after release. The breeding pair attempted to breed twice, and the breeding period was April 3 to May 1, 2020, and May 21 to June 21, 2020. The research period coincided with the breeding period, and information on nest start and end dates, tree species used, and topography was collected through field surveys. Additionally, the distance of each nest to human dwellings, roads, and UCIRC was evaluated. The start of breeding was defined as the moment of egg-laying observed in the nest, and the end was defined as when the eggs were eaten by predators or when the nest was abandoned, confirmed through observations from a higher vantage point.

### Selecting analysis coordinates

The coordinates from the GPS transmitter attached to the breeding pairs were used to understand the behavioral patterns of nest building and egg laying. The location GPS logger(WT-300, KoEco Inc., Korea) is currently used extensively in Korea and has been used in a study comparing the home range of mallards and spot-billed ducks [23] and a study on the home range and habitat use of northern pintails [24]. This backpack type device weighs only 30 g, less than 3–5% of the ibis weight, and gives a minimum limit on its flight [25]. The coordinates were received through the WT-300 12 times a day, at 2-h intervals starting from 00:00. When errors occurred, such as no reception of location information or the coordinates were received twice at 10-minute intervals, the values were designated as null and excluded from the analysis. The valid coordinate values were converted into SHP files using ArcGIS to analyze the home range, movement status, and habitat characteristics of the used areas.

### Home-range estimation

Home-range analysis was performed using minimum convex polygon (MCP; [26]) and kernel density estimation (KDE; [27]) methods based on location information received during the nesting season. MCP is a method for estimating the home range of a species by connecting the outermost points of the collected data [28].

KDE uses the kernel function, one of the nonparametric methods of density estimation, which uses the density distribution graph obtained based on the location coordinates of wildlife and represents it as a line-like curve [29, 30]. Because 95% and 50% KDEs represent the general home range and core habitat, respectively [31], it was used in this study.

A coordinate count of ≥ 50 values is recommended for analysis [29], and because both breeding pairs had ≥ 300 coordinates, it was deemed appropriate for home-range analysis. Additionally, in KDE, appropriate smoothing parameters need to be selected because different parameters lead to different results in terms of shape, variability, and data trends [29, 32, 33]. In This study, we used standard multivariate normalization (h_ref), a widely used methodology known as Silverman’s rule of thumb [27, 34, 35]. The analysis was performed using Home Range Tools 2.0, an extension of ArcGIS 10.2 (ESRI Inc.), and the respective smoothing parameter values were calculated using Animal Space Use 1.3 Beta [36]. Individual occurrence locations were not shown when plotting home ranges because it is a protected species.

### Habitat topography

An analysis was conducted using a 5 m resolution Digital Elevation Model (DEM) based on a 1:5,000 scale topographic map provided by the National Geographic Information Institute to examine the elevation, slope, and aspect of the areas active during the breeding period. The boundary was determined by creating the MCP that combined the coordinate values from the first and second breeding periods of two individuals, using the MCP as a boundary to exclude areas that were included despite no activity. Analysis was performed using QGIS 3.28, with elevation measured in 25 m increments, aspects including flat and eight directions, and slope gradient divided into 5° intervals. the eight directions consist of north, northeast, east, southeast, south, southwest, west, and northwest according to azimuth.

### Habitat characterization

To analyze land use types by distance from the nest, we overlaid land cover maps with occurrence points and categorized the results according to breeding season and land use by distance. In addition, the crested ibis generally use paddy fields, ridges between paddy fields, fields, grassland, shallow rivers, and streams as foraging areas [37, 38], and it was found in field surveys that they use cemeteries when foraging areas are difficult to access because of disturbances. Thus, the number and area of foraging sites used by breeding pairs were determined and analyzed to determine the size of foraging sites required for reproduction. The analysis was based on the results of the home range, and cumulative coordinate distribution distance to set the range. Furthermore [14], reported an average breeding season foraging distance of 563 m centered on a nest, a similarity we intend to research further. We calculated the area and coordinate count of land cover types within the specified range, based on the land cover classification. The land cover types considered for use as feeding areas include paddy fields, fields, natural grassland, cemeteries, lakes(including reservoirs), and inland wetlands. Among the 22 items of middle divisions land cover map [39], Artificial grassland and inland water are related to cemeteries, rivers, and lakes, which are the main feeding grounds for ibis, so two items were further subdivided to create a land cover map classified into 25 items (Fig. 1).

Both analyzes were based on when the ibis was non-flight. The standard for distinguishing between flight and non-flight was based on the speed (km/h) received through GPS device, and when the standard speed was 2 m/s or less [40], it was considered non-flight. The definition of behavior was based on typical behavior of ibises in the land cover type where the emergence point was located (e.g. feeding activity in paddy fields), which was based on previous studies and repeated monitoring of breeding individuals.

## Results

### Nest-building environment

Examination of the characteristics of the nests constructed in the two attempts in terms of topography and distance from environmental factors (Table 1) revealed that red pine(*P. densiflora*) was used as a nesting tree during both attempts. However, the nest was constructed on drooping branches in the first attempt, and it was constructed on the trunk in the second attempt. The first nest was located at an elevation of 37 m and a slope of 24°, 24 m from the nearest dwelling, 141 m from a two-lane road, and 2,240 m from the UCIRC. The second nest was built on a west-facing site at an elevation of 33 m and a slope of 33°, 40 m from the nearest house, 14 m from a two-lane road, and 2,070 m from the UCIRC. In the second nest, the main stem was used compared with the branches in the first nest, and it was positioned closer to the road.

**Table 1:** Breeding period and location information

Legend	First nest	Second nest
Period	April 03–May 01, 2020	May 21–June 21, 2020
Nest location tree	*P. densiflora*	*P. densiflora*
Elevation(m)/Slope(°)/Aspect	37/24/East	33/33/West
Distance from nearest human dwelling	24 m	40 m
Distance from a two-lane road	141 m	14 m
Distance from the UCIRC	2,240 m	2,070 m

### Analysis coordinate selection

The coordinate values received for each individual in the breeding pair(Table 2). The duration of the first nest was 29 days, and 348 coordinates were predicted to be received, but for UPO1918, 355 values were received, and 10 nulls occurred, resulting in 345 valid coordinates. For UPO1938, 352 values were received and 4 nulls were encountered, resulting in 348 valid coordinates, the same as expected. The duration of the second nesting was 32 days, for which 384 coordinates were predicted to be received. However, for UPO1918, 387 coordinates were received, and 6 null values occurred, resulting in 381 valid coordinates, and for UPO1938, 386 coordinates were received and 2 null values occurred, resulting in 384 valid coordinates.

**Table 2:** Number of coordinates received from individual ibis

ID	Sex	Age	First breeding					Second breeding				
			Tracking period	Expected value	Obtained values	Valid	Null	Tracking period	Expected value	Obtained values	Valid	Null
UPO1918	Male	Adult	April, 03–May, 01, 2020 (29 days)	348	355	345	10	May 21–June 21, 2020 (32 days)	384	387	381	6
UPO1938	Female	Adult			352	348	4			386	384	2

### Home range

**Fig. 2:**
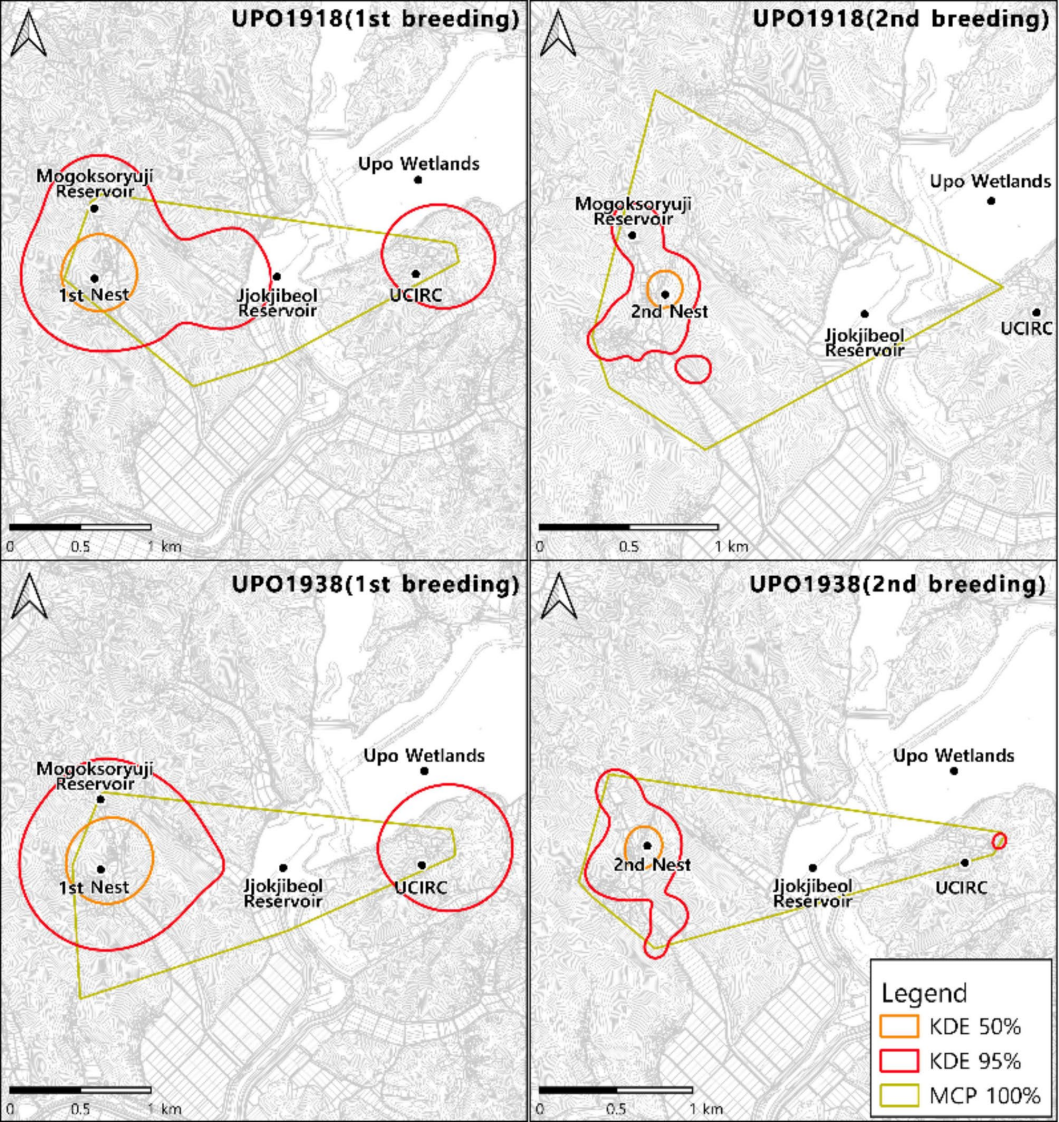
Home range analysis results using MCP and KDE(left: First breeding, right: Second breeding)

Table 3; Fig. 2 illustrate the home ranges and core habitats during the first and second nesting periods. During the first nesting period, the home range measured using MCP was 2.222 km² for UPO1918 and 2.330 km² for UPO1938, encompassing the Mogoksoryuji reservoir, UCIRC, and the areas in between. For the KDE analysis, UPO1918 had a 2.080 km² (95%) and 0.228 km² (50%) range with the core habitat forming a circle centered on the nest.

During the second breeding period, the MCP range for UPO1918 expanded to 2.425 km², while for UPO1938, it contracted to 1.777 km². For the KDE analysis, upo1918 and upo1938 were 0.347km2 and 0.449km2 at 95% KDE, respectively, and 0.032km2 and 0.053km2 at 50% KDE. Compared to the first breeding season, the use area of the UCIRC and the agricultural lands around the Jjokjibeol Reservoir decreased, and the home range was concentrated around the nest.

**Table 3:** Estimated home range of ibis using MCP and KDE(Unit:km^2^)

ID		MCP 100%	Fixed-kernel	
			95%	50%
First breeding	UPO1918	2.222	2.080	0.228
	UPO1938	2.330	2.085	0.296
Second breeding	UPO1918	2.425	0.347	0.032
	UPO1938	1.777	0.449	0.053

### Topographical features

**Fig. 3:**
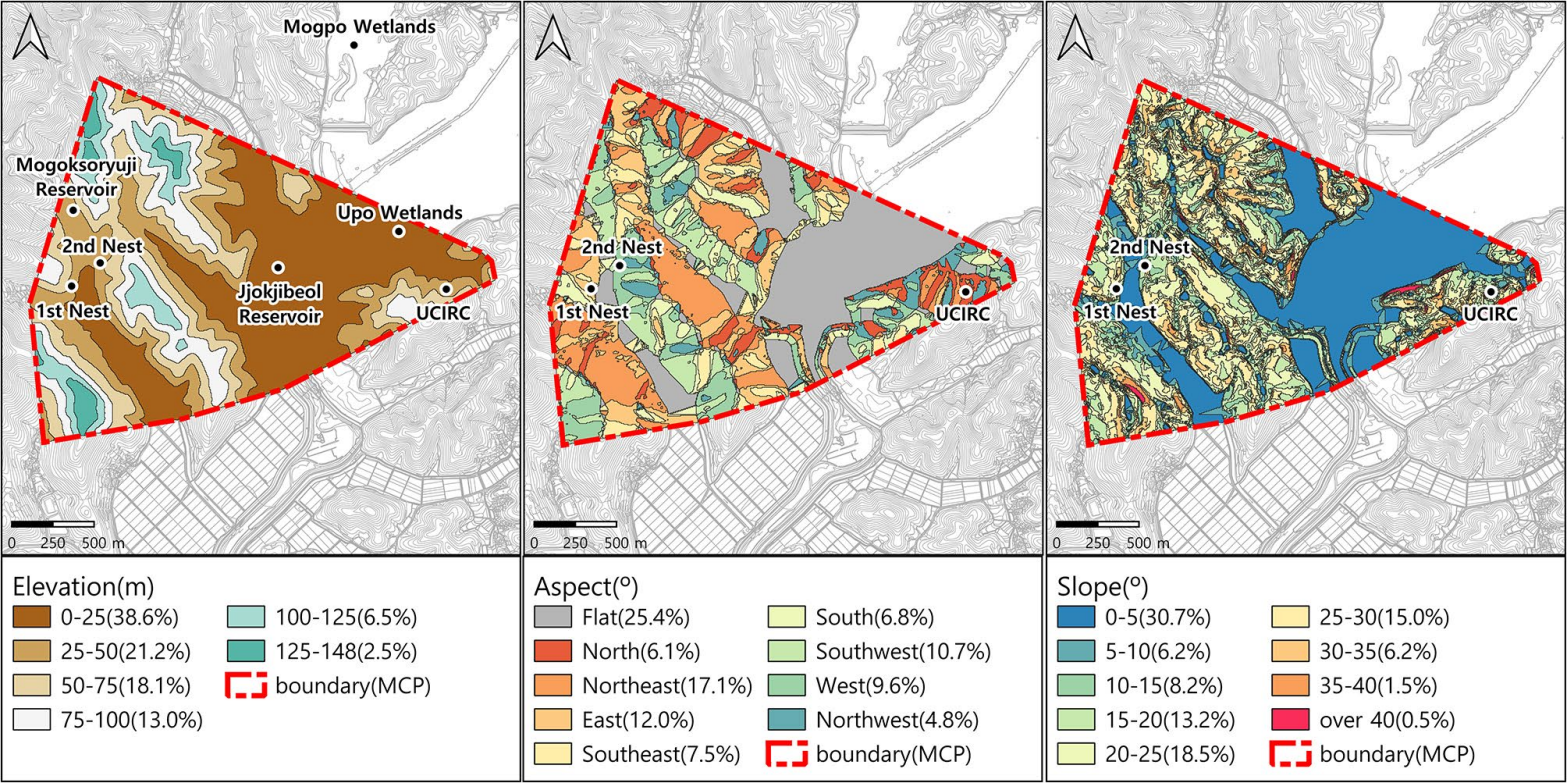
Elevation, Aspect, and Slope analysis results (The boundary area is 3.57 km^2^, and the value in parentheses for each grade indicates the ratio to the boundary area)

Within the boundary, most residential and paddy areas fall within the low elevation range of 5–25 m, making up the largest area at 38.6% of the total (Fig. 3). The 25–50 m range, which includes forest edges and fields, accounts for 21.2% of the activity range, with the Crested Ibis mainly engaging in feeding and resting within the 5–50 m range. The area from 50 to 150 m is mostly forested, with minimal usage by the ibis. Most of the lowland areas were flat and constituted the largest expanse, while the forests showed a diverse distribution of aspects. The nesting site was located on a steep slope at the forest edge of the lowland, between residential areas and the forest.

### Land use by activity distance

Table 4 shows the land use and distances from the nest during the twice of nesting period. In the first breeding period, 14 out of 25 land cover types were identified, with significant usage of coniferous forests (323 coordinates), artificial barren(170), other grasslands (50), residence facilities (44), paddy fields (42), fields (12), and commercial facilities (15) out of the total 682 coordinates. The prevalence of coordinates in coniferous forests is attributed to nest creation in red pine within the forest, used for perching, waiting during alternation, and resting. The land cover type, excluding paddy fields, fields, cemeteries, inland wetlands, and reservoirs, which are considered feeding areas, is believed to be due to the land cover surrounding the nest and covering the UCIRC. The UCIRC, perceived as a safe location due to nesting and a continuous food supply, likely attracted visits for rest and feeding activities. During the maintenance of the second nest, similar to the first nesting period, the crested ibis tended to rest in nest trees and adjacent coniferous trees, primarily red pine, for foraging activities in nearby paddy fields or fields. This was confirmed not only by the results of the analysis but also by repeated ibis monitoring.

Over 50% of the coordinates occurred within 50 m throughout the two nest maintenance periods, the highest usage frequency in the coniferous forests where the nest was constructed. For activities from 50 m to 500 m, the use of paddy fields and fields increased in frequency. The range of 500 to 2,000 m suggests movement between the nest and the UCIRC, while the use of coniferous forest and artificial barren areas beyond 2,000 m indicates that the movement has reached the UCIRC. However, during the second nesting period, 60.0% (456 coordinates) occurred within 50 m. Compared to the first period, the rate within 500 m was higher in the second period and coordinates over 2,000 m decreased significantly. This is likely influenced by the improved feeding environment and previous nest failure experience.

**Table 4:** Analysis of activity based on the distance from the nest (miscellaneous: less than 10 of each classification)

Legend			Distance(m)								Total
			0–50	50–100	100–200	200–300	300–400	400–500	500–2000	> 2000	
**First** **breeding**	**Coniferous forest**	**Number**	183	4	20	74	-	19	10	13	323
		**Ratio**	26.8	0.6	2.9	10.9	-	2.8	1.4	1.9	47.4
	**Artificial barren**	**Number**	143	1	-	-	-	-	-	26	170
		**Ratio**	21.0	0.1	-	-	-	-	-	3.8	24.9
	**Other grassland**	**Number**	5	1	4	1	-	-	18	21	50
		**Ratio**	0.7	0.1	0.6	0.1	-	-	2.6	3.1	7.3
	**Residence facility**	**Number**	40	4	-	-	-	-	-	-	44
		**Ratio**	5.9	0.6	-	-	-	-	-	-	6.5
	**Paddy field**	**Number**	2	33	5	-	-	-	2-	-	42
		**Ratio**	0.3	4.8	0.7	-	-	-	0.3	-	6.2
	**field**	**Number**	-	1	4	2	-	-	5	-	12
		**Ratio**	-	0.1	0.6	0.3	-	-	0.7	-	1.8
	**Commercial facility**	**Number**	12	1	2	-	-	-	-	-	15
		**Ratio**	1.8	0.1	0.3	-	-	-	-	-	2.2
	**Miscellaneous**	**Number**	2	2	6	-	-	4	5	7	226
		**Ratio**	0.3	0.3	0.9	-	-	0.6	0.7	1.0	3.8
	**Total**	**Number**	387	47	41	77	-	23	40	67	682
		**Ratio**	56.7	6.9	6.0	11.3	-	3.4	5.9	9.8	100.0
		**Cumulative ratio**	56.7	63.6	69.6	80.9	80.9	84.3	90.2	100.0	100.0
**Second** **breeding**	**Coniferous forest**	**Number**	417	27	47	50	31	23	10	1	606
		**Ratio**	54.9	3.6	6.2	6.6	4.1	3.0	1.3	0.1	79.7
	**Paddy field**	**Number**	1	-	25	16	-	1	-	-	43
		**Ratio**	0.1	-	3.3	2.1	-	0.1	-	-	5.6
	**Traffic facility**	**Number**	25	1	2	-	-	-	-	-	28
		**Ratio**	3.3	0.1	0.3	-	-	-	-	-	3.7
	**field**	**Number**	6	7	5	4	-	4	-	-	26
		**Ratio**	0.8	0.9	0.7	0.5	-	0.5	-	-	3.4
	**Other grassland**	**Number**	2	4	2	-	2	6	1	2	19
		**Ratio**	0.3	0.5	0.3	-	0.3	0.8	0.1	0.3	2.5
	**Artificial barren**	**Number**	2	-	-	8	-	-	-	2	12
		**Ratio**	0.3	-	-	1.1	-	-	-	0.3	1.6
	**Miscellaneous**	**Number**	3	2	6	3	6	5	1	-	26
		**Ratio**	0.4	0.3	0.8	0.4	0.8	0.7	0.1	-	3.4
	**Total**	**Number**	456	41	87	81	39	39	12	5	760
		**Ratio**	60.0	5.4	11.4	10.7	5.1	5.1	1.6	0.7	100.0
		**Cumulative ratio**	60.0	65.4	76.8	87.5	92.6	97.8	99.3	100.0	100.0

The miscellaneous first breeding lists are traffic facility(7), cemetery(5), reservoir(5), public facility(5), inland wetland(2), mixed forest(1), and broad-leaved forest(1). The second breeding miscellaneous lists are residential facility(7), reservoir(6), cemetery(4), commercial facility(3), inland wetland(3), and mixed forest(3)

### Feeding ground area and frequency of use

**Fig. 4:**
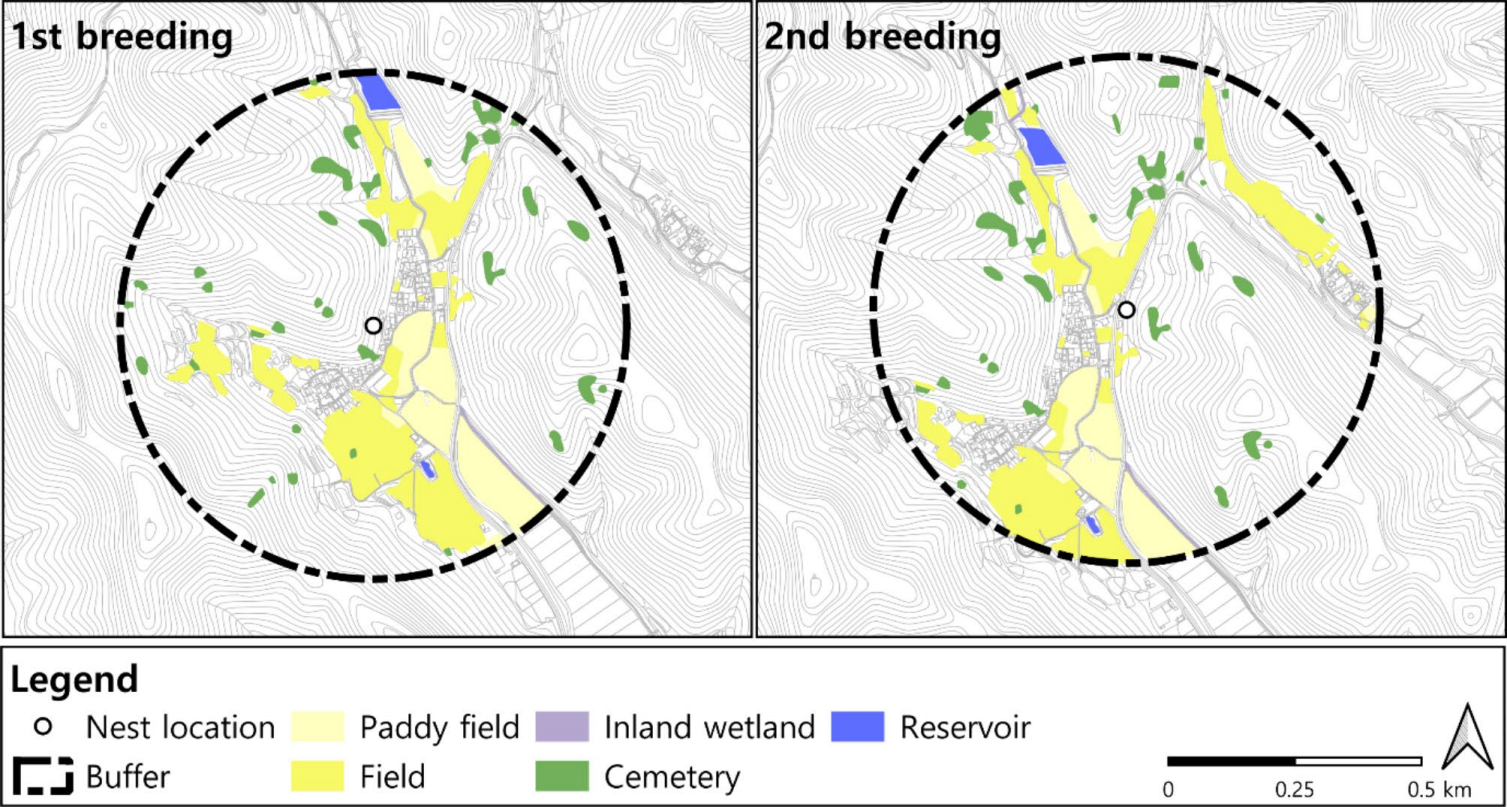
Distribution of feeding land cover types within 500 m of nest sites

Table 5; Fig. 4 display the frequency of use of foraging sites within 500 m, where over 90% of all use is concentrated, based on activity patterns during the secondary nesting period after adaptation from the first nest’s failure. There was no significant change in the feeding area according to land cover type between the first and second breeding periods, with a maximum difference of about 4,600 m². However, the difference in the number of times the fields were used reached 19 times. Fields and cemeteries had low usage relative to their area, while Paddy fields had high usage relative to their area.

**Table 5:** Area and number of uses by feeding ground type

Legend	First breeding		Second breeding	
	Area(m^2^)	Number	Area(m^2^)	Number
**Paddy field**	48,264.3	40	43,763.2	43
**Field**	84,695.20	7	89,307.10	26
**Cemetery**	23,264.0	4	24,803.0	4
**Inland wetland**	1,153.7	-	1,662.0	3
**Reservoir**	4,309.8	4	4,321.5	6

## Discussion

The Crested Ibis, once widespread in East Asia, has suffered a dramatic population decline due to habitat degradation, hunting, changes in agricultural practices, and lack of protective policies [9]. This species is classified as Endangered (EN) on the IUCN Red List, listed on CITES Appendix I, designated as Endangered Wildlife Class II in South Korea, and regionally extinct (RE) on the National Red List, emphasizing its critical conservation status.

In China, before the 1950s, the ibis coexisted near human settlements. However, from 1950 to 1979, it moved to less disturbed areas, and from 1980 to 2000, it further retreated to remote regions for nesting and foraging [41]. The loss of mature trees since the 1950s significantly impacted its population [42]. The last remaining population in the Qinling Mountains, which provides suitable nesting trees and paddies, suggests that reverting to traditional agricultural practices could be vital for current conservation strategies [43]. China has successfully increased the ibis population through captive breeding, habitat restoration, and community support, growing from critically low numbers in the 1980s to a stable population of about 1,000 individuals and over 2,000 worldwide, including captive-bred and reintroduced populations in China, Japan, and Korea [10, 44].

In Korea, restoration efforts began in the late 2000s with artificial incubation and breeding, followed by releases into the wild. Despite progress in breeding and captive propagation, research on the behavior and habitat use of released individuals remains essential. Studies should focus on habitat evaluation, breeding success factors, and natural reintroduction [41, 45–50]. Since the first release in 2019, about 40 individuals have been released annually, bringing the total number of individuals released into the wild to over 100. However, there is a lack of studies on their habitat use and home range, which are crucial for effective habitat management.

The terrain of nesting areas included low-elevation, flat areas within 50 m and slopes less than 5°, encompassing residential, agricultural, wetland, and forest margins. Nests were primarily located in red pine trees in lowland forests. The ibis initially coexisted near human settlements but moved away due to agricultural practices such as fertilizer and pesticide use, winter field drying, and hunting [41]. However, conservation activities have brought them closer to human settlements again [46, 51]. They prefer nesting in high trees in gentle forests near foraging grounds [49, 52].

Home range analysis using MCP and KDE, the area of the second breeding season decreased compared to the first breeding season and was concentrated near the nest. In China, the average breeding season home range of the crested ibis in the Shaanxi Province was MCP 100% 1.008 ± 0.498 km² and KDE 50% 0.438 ± 0.215 km² [14]. In Japan, during the non-breeding season from July to September before crop harvesting, the daily average MCP 100% area was 0.819 ± 0.631 km² [53]. Compared to the Chinese and Japanese populations, the MCP was wider, which may be related to the disadvantages of the MCP, but may also be due to factors such as lack of experience, proximity to the UCIRC, which received adaptation training, and the search for safe places for food and rest. Meanwhile, KDE was smaller, which is presumed to be because the nest was built in the upper part of the valley rather than in an open area in terms of topography, and food was supplied to the paddy fields near the nest. In other words, it is consistent with research showing that gender, release method, season, and individual characteristics influence establishing the home range [54]. Our findings suggest that home ranges may have been overestimated or underestimated due to autocorrelation and distant points. Despite this, initial research is necessary to compare the home ranges of Japanese and Chinese individuals previously studied and identify core areas through multiple analysis methods [55].

Meanwhile, various methods are available to estimate home ranges using GPS-collected data, including AKDE [56], KDE, MCP, and LoCoH [57]. Among these, AKDE is known for its higher accuracy [58]. This study utilized the traditional methods of KDE and MCP. MCP tends to overestimate habitat range [59, 60], while KDE tends to underestimate it due to autocorrelation [61]. However, MCP can provide reliable estimates when there are more than 100 data points [62], and KDE is considered the next best method after AKDE [58, 63]. Additionally, it is the optimal method for comparing crested ibis research in other countries.

The movement distances from nests were predominantly within 50 m, with most coordinates distributed within this range. The usage of coniferous forests for nesting and surrounding areas for resting and foraging was common, aligning with other studies [14]. The high frequency of use of the UCIRC for resting and feeding indicates that it is perceived as a safe area with abundant food resources. However, differences in habitat usage between the first and second nesting periods suggest an adaptation to available resources and surrounding environments.

Challenges in foraging during the breeding season due to agricultural activities necessitate maintaining rice fields as feeding grounds during critical periods. Collaboration with local governments and implementing ecosystem service payment schemes could help preserve these habitats. Reducing pesticide and fertilizer use in paddy fields and creating waterlogged fields in winter could enhance habitat stability [64].

In conclusion, ensuring the stability and growth of the Crested Ibis population requires comprehensive community-based conservation measures. This includes active local community participation and benefits, alongside habitat management strategies tailored to the specific needs of the ibis during breeding and non-breeding seasons. Future research should focus on detailed studies of emerging breeding individuals to better understand home range and movement patterns during critical periods. Additionally, establishing baseline data from this study’s behavioral comparisons and nesting attempt analyses is vital for guiding future research and informing long-term conservation strategies, helping to monitor trends and improve habitat and species management over time.

## Conclusion

The reintroduction of the Crested Ibis in Korea has shown promising results, with the first breeding pair (UPO1918 and UPO1938) forming in April 2020. Home range analysis using MCP and KDE revealed variations compared to individuals in China and Japan, attributed to inexperience and differences in food supply and analysis methods. Predominantly, more than half of the coordinates were within 50 m of the nest, with coniferous forests used for resting and paddy fields as the main feeding areas within 500 m. However, even during the breeding season from April to June, garlic and onions are grown in the rice fields, and there was a tendency for some individuals to travel farther to forage.

In Changnyeong-gun, the extensive cultivated land provides a suitable habitat for the released Crested Ibis, but the breeding season presents challenges due to garlic and onion farming before converting to paddies. Ensuring stable egg laying and brooding requires further research on the average home range and movement distance during the breeding season. Effective maintenance strategies for paddies, the primary feeding grounds in the core habitat, are essential for the continued success of the Crested Ibis reintroduction program.

## Data Availability

The data that support the findings of this study are available from the authors but restrictions apply to the availability of these data, which were used under license from the Upo Crested Ibis Restoration Center for the current study, and so are not publicly available. Data are, however, available from the authors upon reasonable request and with permission from the Upo Crested Ibis Restoration Center.

## Abbreviations

UCIRC: Upo Crested Ibis Restoration Center
P. densiflora: Pinus densiflora
